# Paradoxical tunnel enlargement after ACL reconstruction with hamstring autografts when using β-TCP containing interference screws for tibial aperture fixation- prospectively comparative study

**DOI:** 10.1186/s12891-017-1757-0

**Published:** 2017-09-16

**Authors:** Joon Ho Wang, Eun Su Lee, Byung Hoon Lee

**Affiliations:** 1 Department of Orthopaedic Surgery, Samsung Medical Center, Sungkyunkwan University School of Medicine, Seoul, 06351 South Korea; 2 Department of Health Sciences and Technology, SAIHST, Sungkyunkwan University, Seoul, South Korea; 3 Department of Medical Device Management and Research, SAIHST, Sungkyunkwan University, Seoul, South Korea; 4 Department of Orthopaedic Surgery, Dongbu Jaeil Hospital, Seoul, Republic of Korea; 5 Department of Orthopaedic Surgery, Kang-Dong Sacred Heart Hospital, Hallym University Medical Center, Gil-dong, Seoul, 134-701 South Korea

**Keywords:** ACL, Tunnel enlargement, Interference screw, Plla, β-TCP, Hamstring autograft

## Abstract

**Background:**

Tibial aperture fixation with a bioabsorbable interference screw is a popular fixation method in anterior cruciate ligament reconstruction (ACLR). An interference screw containing β-tricalcium phosphate (β-TCP) to improve bony integration and biocompatibility was recently introduced. This study aims to compare the clinical outcomes and radiological results of tunnel enlargement effect between the 2 bioabsorbable fixative devices of pure poly-L-lactic acid (PLLA) interference screws and β-TCP-containing screws, for tibial interference fixation in ACLR using hamstring autografts.

**Methods:**

Eighty consecutive patients who had undergone double-bundle ACLR between 2011 to 2012 were prospectively reviewed and randomly divided into two groups based on the type of tibial interference screw: 28 were assigned to the pure PLLA screw group (Group A), while the other 29 were assigned to the β-TCP-containing screw fixation group (Group B). Clinical evaluations and radiological analyses were conducted in both groups with a minimum 2- year follow-up.

**Results:**

There was no significant difference in subjective or objective clinical outcome between the 2 groups. In radiological analyses, the use of a β-TCP-containing screw reduced tunnel widening in the portion of the tunnel with screw engagement compared to the pure PLLA screw, while the use of a β-TCP-containing screw resulted in greater tunnel enlargement in the proximal portion of the tunnel without screw engagement than use of a pure PLLA screw.

**Conclusion:**

Use of a β-TCP-containing interference screw in tibial aperture fixation reduced tunnel enlargement in the vicinity of the screw, whereas greater enlargement occurred proximal to the screw end relative to use of a pure PLLA interference screw. These paradoxical enlargements in use of β-TCP containing screws suggest that for reducing tunnel enlargement, the length of the interference screw should be as fit as possible with tunnel length in terms of using soft grafts.

***Level of Evidence:*** II, Prospectively comparative study.

**Trial registration:**

Retrospectively registered with ClinicalTrials.gov. (NCT02754674), Date of trial registration: February 10, 2016.

**Electronic supplementary material:**

The online version of this article (10.1186/s12891-017-1757-0) contains supplementary material, which is available to authorized users.

## Background

Interference screws are commonly used for fixation in orthopedic surgery, especially in tibial aperture fixation for ACL reconstruction. Biodegradable cannulated interference fixation screws made of poly-L-lactic acid (PLLA) were introduced in the early 1990s to improve postoperative imaging, decrease stress shielding, avoid graft laceration, and make revision easier [1, 2]. These screws have a fixation strength equivalent to that of metal screws [3–5]. Bioabsorbable fixation devices offer advantages over metallic devices in that they can be substituted for bone contrary to metal screws; bone tunnel remodeling is not possible when metal interference screws are in place.

However, there are concerns that explanted pure PLLA biodegradable interference screws may not degrade, and that replacement with bone may be incomplete [1, 6–8], and the incidence of tunnel enlargements following the use of resorbable devices has been shown to be higher than metallic devices [9–11]. A variety of biodegradable screws with different material compositions have therefore been introduced. Not all bioabsorbable materials have the same compositions, absorption rates, or tissue reactions. Some of the bioabsorbable materials commonly used for interference screws are polyglycolic acid (PGA), polylactic acid (PLA), polyparadioxanone (PDS), polymers of PGA/PLA, and various stereoisomers of lactic acid (PDLA).

The Matryx® (Linvatec Corp, Largo, FL, USA) screw, made of 96 L/4D poly lactic acid (PLA) and 30% β-tricalcium phosphate (β-TCP), was introduced to enhance osteoconduction and to be more biocompatible with bone than existing screw materials. However, to the best of our knowledge, the effect of this screw against tunnel widening effect by osteoconduction and bony integration around tunnels has not been investigated in a clinical setting. Therefore, we sought to investigate the clinical advantages of β-TCP containing biodegradable screws under their theoretical advantages.

We hypothesized that use of β-TCP-containing interference screws would improve coaptation of the graft to the tunnel with replacement of the degraded screw by new bone formation, and result in better tunnel remodeling by preventing tunnel widening. The purpose of this study was to evaluate whether use of a β-TCP-containing interference screw resulted in superior clinical and radiological outcomes (esp, the preventive effect on tunnel enlargement during in vivo degradation within the tunnel) in ACL reconstruction using autologous hamstring grafts compared to a commercially available pure PLLA biodegradable screw.

## Methods

### *Level of Evidence* II, prospectively comparative study

**Fig. 1:**
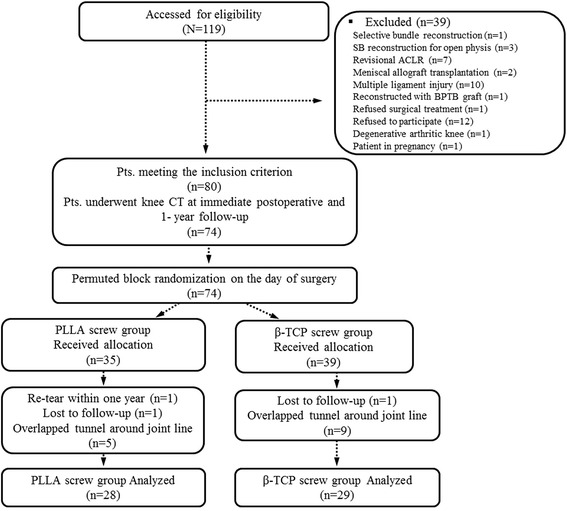
Patient flowchart. (ACLR, anterior cruciate ligament reconstruction; SB, single bundle; BPTB, bone-patellar tendon-bone; CT, computed tomography)

From February 2011 to June 2012, a total of 119 patients with ACL injury were enrolled in this study. Inclusion criteria were patients who underwent a primary anatomical double bundle ACL reconstruction with tibial aperture fixation by Bioscrew® (Linvatec Corp, Largo, FL, USA), which is made of 100% poly-L-lactic acid (PLLA) or Matryx® (Linvatec Corp), which is made of 70% 96 L/4D poly lactic acid (PLA) & 30% β-TCP, and who were at least 20 years of age; patients younger than 20 years of age were treated on the basis of adolescent ACL and single-bundle reconstruction with no interference screw placed in the tibial tunnel if open physis. Of these 119 patients, 39 were excluded for the following reasons: single-bundle reconstruction (4), revisional ACL reconstruction (7), combined surgeries such as meniscal allograft transplantation (2), any previous ligament surgery or bony procedure in the area of graft attachment (10), degenerative arthritic knee (1), pregnancy (1), and other reasons (14) (Fig. 1). After exclusion, 80 patients underwent DB-ACLR according to a trial comparing two different interference screws. Eighty patients were randomly assigned to either the PLLA screw group or the β-TCP screw group on the day of surgery using permuted block randomization [12], and 74 underwent knee computed tomography (CT) immediately and 1-year after the operation (Fig. 1).

To assess differences in tibial tunnel widening according to fixation device, the cross sectional areas at four locations were compared between the two groups on the immediate postoperative and 1- year follow-up CT images. All operations were performed by a single surgeon (J.H.W.) experienced in ACLR using both the transportal and outside-in techniques. All patients underwent CT examination twice on the same CT device using the same protocol within 3 days after the initial surgery and at the postoperative 1-year follow-up. The current study received Institutional Review Board approval from our institution (Samsung Medical Center 2010–08-116) before study onset, and our protocol was also approved. Informed consent was obtained from all participants (ClinicalTrials.gov identifier: NCT02754674).

#### Surgical technique and rehabilitation

All operations were performed using an arthroscopic-assisted technique. Femoral and tibial tunnels were created in the centers of the respective anatomic insertions. Semitendinosus and gracilis tendons were harvested from the affected limb. The tunnels were prepared to be the same size as the graft, and the femoral tunnel was drilled to this diameter by matching the drill and/or dilator to the graft size while accounting for the size of the native insertion site. The drill diameter for the femoral and tibial tunnels was determined based on the diameter of the prepared graft. Grafts were inserted retrograde via the tibial tunnel into the femoral tunnel, and fixed with a cortical suspension system using the shortest possible loop (10 to 15 mm) to ensure maximal contact between the graft and the tunnel walls on the femoral side, and with bioabsorbable interference screws with a post tie on the tibial side for all cases.

Graft was normally trimmed to retain its original size with a length of 26 to 30 cm. Then, it was folded and sutured together with the use of no. 1 absorbable sutures to form two triple-strand grafts. A 6-stranded graft, composed of triple semitendinosus (6.5 to 9.5 mm) for the anteromedial (AM) bundle and triple gracilis (4.5 to 7 mm) for the posterolateral (PL) bundle, was created for each group.

Anatomic tibial insertion sites of both bundles were marked with an ArthroCare device (ArthroCare, Sunnyvale, CA, USA), and the tip of the guide was aimed at the centers of the AM and PL bundle remnant tibial insertion sites. A 3.2-mm guide pin was inserted into the bases of the AM and PL tibial insertion sites. The AM and PL tibial tunnels were then drilled with a cannulated drill. Bioscrew® or Matryx® (length 30 mm) with a diameter 1 mm larger than the tunnel diameter was used for tibial aperture fixation at 0^o^ of knee flexion and applying a 30–35 N tension to the graft using the dedicated tie tensioner [13], and an additional extracortical screw with a spike washer was applied. Interference screws eccentrically compress the graft against the wall of the tibial bone tunnel. The type of all screws was blinded at insertion to surgeon for randomized allocation.

All patients began active quadriceps isometric exercise and active range-of-motion exercise immediately after surgery. Four to 5 days after surgery, an ACL limited-motion brace was applied, and joint motion exercise was carried out at 15° increments per week. At 4 and 6 weeks after surgery, 90° and 135° of motion, respectively, were allowed. A pair of crutches was used to allow partial weight bearing from 3 days to 6 weeks after surgery. Patients were educated on performing proprioceptive balancing exercise at 3 months after surgery. Return to competitive sports involving jumping, pivoting, or sidestepping was prohibited until 6 months after the reconstruction [14].

#### CT evaluation & measurement

**Fig. 2:**
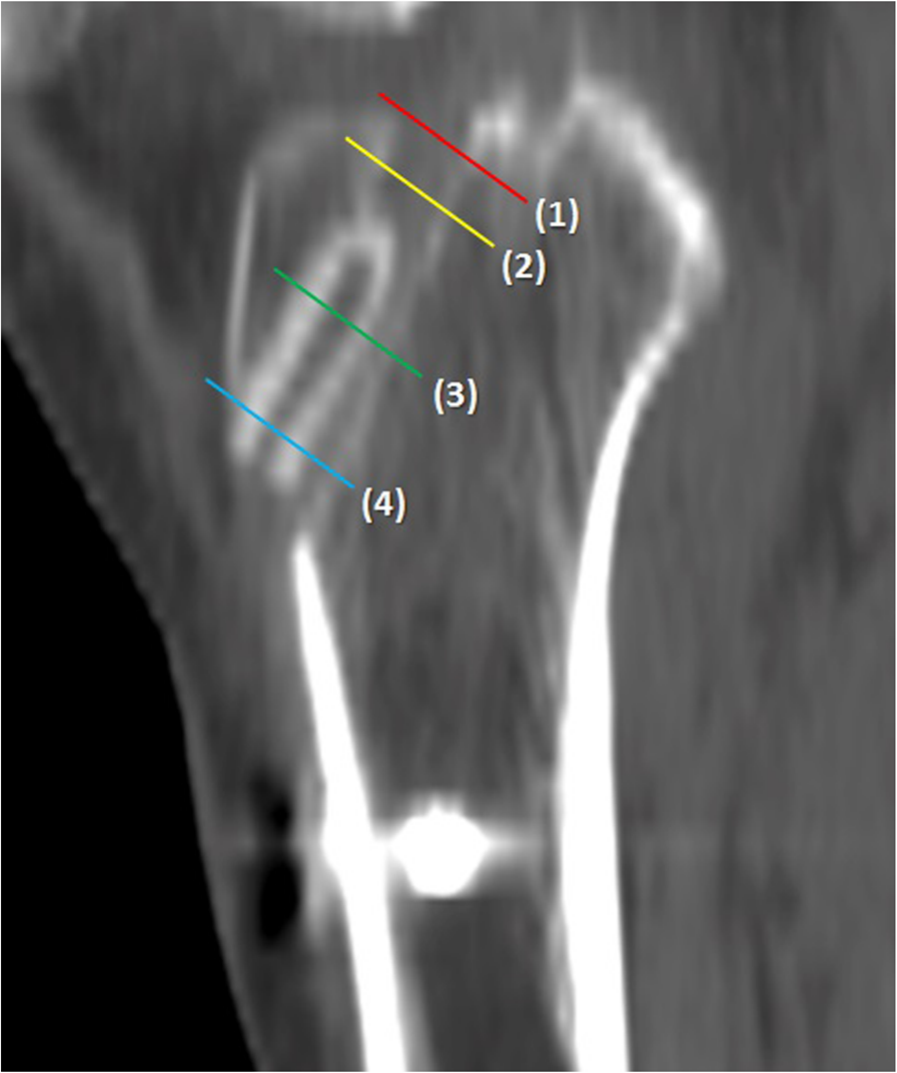
Cross-sectional area was measured at the four cutting levels of tibial tunnel in the plane perpendicular to the long axis of the tunnel: (1) joint line, (2) mid-tunnel, (3) mid-screw, and (4) outlet

**Fig. 3:**
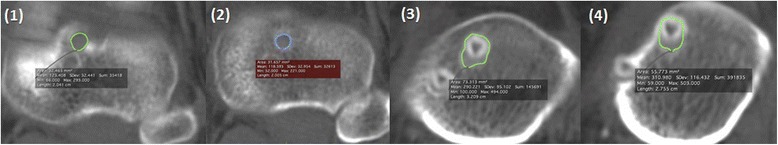
Cross-sectional view and area (mm^2^) measurement of (1) the joint line, (2) the mid-tunnel, (3) the mid-screw, and (4) the outlet cutting level

A CT scanner Light Speed VCT (GE Medical Systems, Milwaukee, WI, USA) was used for all examinations. All patients were placed in a supine position with knee full extension. The collimation was 16 × 0.625 mm, the tube parameters were 120 kVp and 200 mA, and the acquisition matrix was 512 × 512 pixels. Images were processed for multiplane reconstruction, and cross-sectional area (CSA) was measured using Osirix (version 3.5.1; Pixeo, Geneva, Switzerland). We measured CSAs in the plane perpendicular to the long axis of the tibial tunnel using Osirix at the following four cutting levels (Fig. 2): (1) joint line, which was just below the proximal joint line, (2) center level of the tunnel without screw engagement (mid-tunnel), (3) center level of the tunnel with screw engagement (mid-screw), and (4) tunnel aperture outlet. The difference in CSA at each of the four cutting levels was compared between the two groups on immediate postoperative and postoperative 1-year CT scans (Fig. 3). CSA measurements were performed by two training fellows in sports medicine. If the AM and PL tunnels overlapped around the joint line, the CSA of the joint line was excluded from the statistical analysis because of the inability to measure each value separately.

#### Clinical evaluation

Clinical follow-up evaluations were then conducted at 12 and 24 months and made by an independent examiner (ARK). The preoperative evaluation was conducted by the Lysholm knee score, Hospital for Special Surgery (HSS) score, Tegner score, the International Knee Documentation Committee Score (IKDC) [15–17] and a ligament stability assessment by KT-2000® arthrometer (MEDmetric, San Diego, CA) testing with a side-to-side difference of 30 lb. with the knee at 20° of knee flexion. The differences of ligament stability between both legs were calculated by subtracting the laxity measurement of the uninjured knee from the laxity measurement of the injured knee. Intraoperative and postoperative complications were recorded.

#### Reliability and statistical analysis

Two orthopedic surgeons (independent observers) developed and agreed to the measurement methods together; however, they were blinded to each other’s measurements and their previous measurements. They measured the cross-sectional areas at the four cutting levels for all knees twice with an interval of 2 weeks. Reliability of the measurements was assessed by an independent statistician who examined interobserver reliability using the intraclass correlation coefficient. A priori power analysis was performed to determine the sample size using the two-sided hypothesis test at an α level of 0.05 and a power of 0.8. A post hoc power analysis was performed to determine whether the results of our study of 25 cases indicated adequate power, and the power was 85.21%, which was adequate to detect a difference of 1 mm widening [18, 19]. The paired t-test was applied to compare cross-sectional area at the four cutting levels between both groups on immediate postoperative and 1-year follow-up CT scans. *P* < 0.05 was considered significant (95% confidence intervals). The 2-sample t-test was applied to compare the cross-sectional areas of “mid-tunnel” and “mid-screw”. The difference of clinical outcome scales between two groups was evaluated with a 2-sample t test or Mann-Whitney test. Statistical analyses were executed using SAS version 9.3 (SAS Institute, Cary, NC).

## Results

### Demographics

The mean age was 34.7 years (range, 20 to 60 years) and the mean body mass index (BMI) was 24.6 kg/m^2^ (range, 19.1 to 35.2 kg/m^2^). The mean duration of postoperative follow-up was 33 months (range, 24 to 60 months). There were no significant differences between groups in age, gender, BMI, femoral tunneling technique, and tunnel diameter, values of which are shown in Table 1. On immediate postoperative CT, CSA at each cutting level was not significantly different between the two groups in either the AM or PL tunnels. AM and PL tunnel overlap around the joint line was found in five cases in group A and nine cases in group B.

**Table 1:** Patient demographics^a^

	Group A[PLLA screw](*n* = 28)	Group B[β-TCP screw](*n* = 29)	*p* - value
Age, *y*	36.1 ± 8.9	32.7 ± 12.3	0.225
Sex, male/female, *n*	21:7	24:5	0.473
BMI, *kg/m* ^*2*^	24.2 ± 2.6	24.7 ± 3.9	0.848
Femoral tunneling technique, *n* TP: OI	11:17	17:12	0.144
Tunnel diameter (drill size, mm)			
AM	7.5 ± 0.6 (6.5–9.5)	7.4 ± 0.6 (6.0–9.0)	0.387
PL	5.7 ± 0.5 (4.5–7.0)	5.7 ± 0.5 (4.5–7.0)	0.675
Tunnel length (mm)			
AM	39.6 ± 3.9 (34–50)	40.0 ± 2.9 (34–45)	0.661
PL	43.7 ± 3.5 (35–50)	44.8 ± 3.0 (38–50)	0.228

*BMI* body mass index, *TP* transportal, *OI* outside-in

^a^Value are presented as mean ± standard deviation, with range in parentheses

No cases of complete absorption and replacement of the interference screw with new bone were observed at the 1-year follow-up CT in either group. No clinical evidence of any adverse events related to these interference fixation screws was observed, and degenerative radiographic changes were not noted.

### Radiological results (tunnel enlargement)

In both AM and PL tunnels, the average CSAs at the four cutting levels were significantly greater at postoperative 1 year than immediately post-operatively, and the most increased CSAs were observed at the mid-tunnel level (Tables 2 and 3). There were significant inter-group differences in increments of CSAs of the AM tunnel 1 year after surgery at the mid-tunnel and mid-screw cutting levels. At the mid-screw level, mean increase in CSA after 1 year was significantly smaller in group B (β-TCP screw) than in group A (PLLA screw) (16.8% vs. 27.2%, *p* = 0.024). At the mid-tunnel level, the mean increase in CSA after 1 year was significantly larger in group 2 (β-TCP screw) than in group 1 (PLLA screw) (66.9% vs. 40.1%, *p* = 0.028) (Table 2). In PL tunnel analysis, there was no significant difference in average increments of CSAs at the four cutting levels (Table 3). The measurement of CSAs at the four cutting levels showed a good reliability (an appendix is available as a Additional file 1: Table S-1).

**Table 2:** Comparison of the cross-sectional area of the AM tibial tunnel at the four cutting levels between immediate postoperative and postoperative 1 year CT scans

Cutting level		Cross sectional area (mm^2^)			
	Group	Immediate postop.	1YR	Difference	^†^ *P*
Joint line	1	41.5 ± 6.6	54.6 ± 11.3	13.2 (31.7%)	< 0.001
	2	42.0 ± 9.1	57.3 ± 12.1	15.4 (36.6%)	< 0.001
^*^ *P*				0.523	
Mid-tunnel	1	41.8 ± 7.2	58.6 ± 11.6	16.8 (40.1%)	< 0.001
	2	41.9 ± 8.7	70.0 ± 19.5	28.1 (66.9%)	< 0.001
^*^ *P*				**0.028**	
Mid-screw	1	61.0 ± 10.5	77.6 ± 13.3	16.6 (27.2%)	< 0.001
	2	63.3 ± 9.8	73.9 ± 14.2	10.6 (16.8%)	< 0.001
^*^ *P*				**0.024**	
Outlet	1	62.0 ± 10.6	69.2 ± 15.1	7.3 (11.7%)	< 0.001
	2	61.6 ± 11.3	68.4 ± 14.4	6.8 (11.0%)	< 0.001
^*^ *P*				0.833	

1YR; postoperative 1 year

^*^Comparison of the increments in cross-sectional area 1 year after surgery between the two groups at the each cutting level

^†^Comparison of the cross-sectional area between immediate postoperative and postoperative 1 year CT scans

^*^Values of *P* < 0.05 are displayed in bold

**Table 3:** Comparison of the cross-sectional area of the PL tibial tunnel at the four cutting levels between immediate postoperative and postoperative 1 year CT scans

Cutting level		Cross-sectional area (mm^2^)			
	Group	Immediate postop.	1YR	Difference	^†^ *P*
Joint line	1	21.1 ± 6.3	28.8 ± 10.5	7.7 (36.3%)	0.002
	2	24.5 ± 4.1	34.6 ± 7.5	10.1 (41.5%)	< 0.001
^*^ *P*				0.411	
Mid-tunnel	1	23.0 ± 6.6	30.7 ± 11.8	7.7 (33.5%)	< 0.001
	2	25.0 ± 3.8	36.0 ± 10.0	11.0 (44.2%)	< 0.001
^*^ *P*				0.179	
Mid-screw	1	36.6 ± 8.58	42.4 ± 12.8	5.8 (15.8%)	< 0.001
	2	40.0 ± 6.83	46.7 ± 13.3	6.7 (16.8%)	0.003
^*^ *P*				0.734	
Outlet	1	33.3 ± 9.4	34.1 ± 11.0	0.8 (2.5%)	0.445
	2	36.1 ± 8.0	36.6 ± 10.0	0.5 (1.4%)	0.647
^*^ *P*				0.845	

1YR; postoperative 1 year

^*^Comparison of the increments in cross-sectional area 1 year after surgery between two groups at each cutting level

^†^Comparison of the cross-sectional area between immediate postoperative and postoperative 1 year CT scans

### Clinical results

Mean Lysholm score, HSS and IKDC values and KT-2000 measurements values are reported in Table 4. At 2 years, the mean side-to-side difference for anterior displacement at 30° flexion was 2.1 mm (SD ± 1.1 mm) in group A and 1.8 mm (SD ± 1.5 mm) in group B. The mean Lysholm score was 94.4 (SD ± 6.8) in group A and 94.1 (SD ± 6.3) in group B. In group A, the mean IKDC subjective score was 85 (SD ± 11.7) in group A and 86.4 (SD ± 9.5). At the last follow-up, both groups reached a satisfactory pain relief and functional improvements, without significant differences. KT-2000 side-to-side differences (p = n.s.) were similar between the two types of screw fixation. In our experience, the difference of tibial tunnel enlargement did not affect clinical results at 2 years.

**Table 4:** Clinical outcomes

	Group A [PLLA screw] (*n* = 28)	Group B [β-TCP screw] (*n* = 29)	p - value
KT-2000™ side-to-side difference (mm)			
Baseline	4.5 ± 2.2	4.5 ± 2.2	0.964
24 months	2.1 ± 1.1	1.8 ± 1.5	0.334
Lysholm knee score			
Baseline	71.6 ± 21.7	61.9 ± 22.8	0.102
24 months	94.4 ± 6.8	94.1 ± 6.3	0.848
HSS score (/100)			
Baseline	92.1 ± 11.8	86.6 ± 15.7	0.131
24 months	99.4 ± 1.7	99.6 ± 1.9	0.691
IKDC subjective score			
Baseline	58.2 ± 16.2	51.6 ± 19.0	0.157
24 months	85.0 ± 11.7	86.4 ± 9.5	0.610
Tegner score			
Baseline	3.7 ± 1.4	3.8 ± 1.9	0.706
24 months	6.3 ± 1.5	6.3 ± 1.6	0.977

Values are expressed as median ± standard deviation

*ROM* range of motion, *IKDC* International Knee Documentation Committee

## Discussion

The principal findings of our study were as follows: (1) the use of a β-TCP-containing screw reduced tunnel widening in the portion of the tunnel with screw engagement compared to the pure PLLA screw, while (2) the use of a β-TCP-containing screw resulted in greater tunnel enlargement in the proximal portion of the tunnel without screw engagement than use of a pure PLLA screw. These findings might be explained that in the screw-bone contact area, particles during degradation of β-TCP led to less local reactivity or inflammatory responses under their neutralizing effects, whereas they activated osteoclasts and resulted in osteolysis with mechanical stress or synovial fluid influx in proximal portion without screw engagement.

The use of biodegradable interference screws is widely accepted because of their ease of handling and effective fixation. Ideally, a biodegradable interference screw should degrade with minimal or no host-bone reaction. It is reasonable to assume that the faster a material degrades, the earlier the osseous replacement takes place [20, 21]. However, tissue reaction occurs only during and after degradation of the implant [22]. In fact, use of interference screws made of other, more rapidly degrading polymers has been reported to result in collection of fluid at the top of the femoral bone tunnel and significant tunnel widening [23].

In terms of bone-patellar tendon-bone (BPTB) grafts, interference screws have the benefit of reducing the potential space created in bone by the compaction method, which forms the walls of the container [24]. Several MRI studies have showed no signs of container phenomenon or pathologic signals at long-term follow-up. In terms of autologous hamstring tendons, the use for ACL reconstruction has increased in popularity over the recent years [25]. However, even the use of an interference screw with a size matching that of the tunnel diameter will not totally prevent synovial fluid from infiltrating into the tunnel during the biologic transition of thin fibrous tissue into dense fibrous tissue. Practically, although we used only interference screws with a diameter 1 mm larger than the tunnel diameter for tibial aperture fixation in our study, significantly increased CSAs were observed at all four cutting levels at postoperative 1 year in both AM and PL tunnels.

**Fig. 4:**
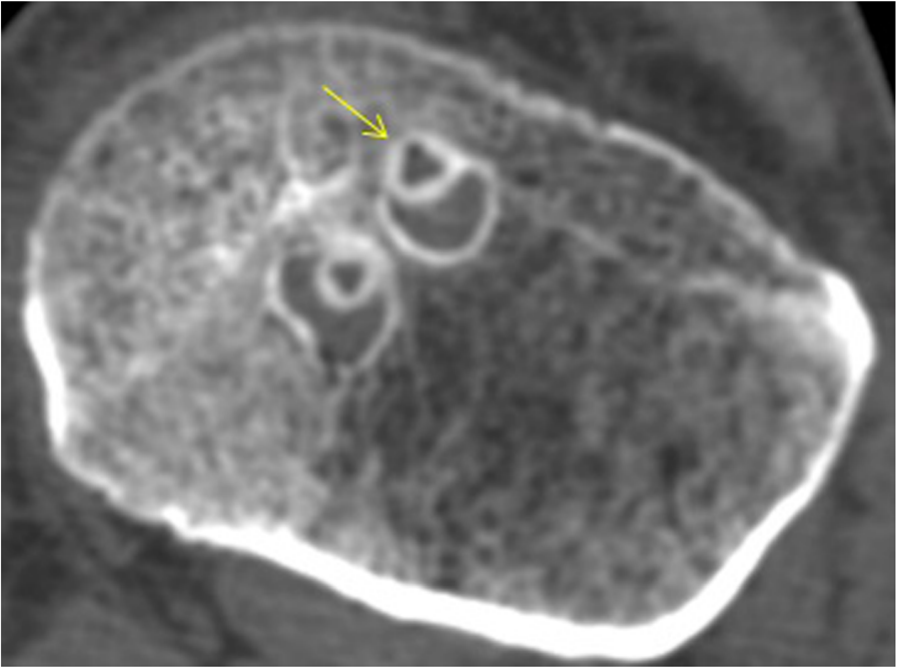
Bony integration was observed in the screw-bone contact area in group B

**Fig. 5:**
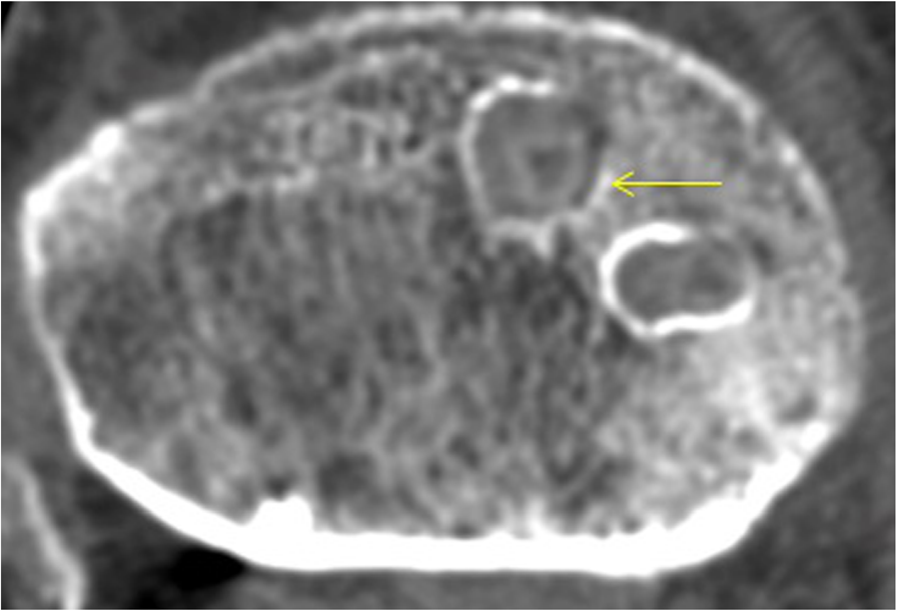
Sclerotic margin without bony integration in the screw-bone contact area was observed in group A

However, our finding of a smaller increase in tunnel widening in the portion of the tunnel with β-TCP screw engagement has demonstrated a better behavior in clinical settings. Our hypothesis was that early degradation and the osteoconduction-promoting effect of β-TCP screws would enhance bony integration and reduce tunnel enlargement. As hypothesized, the tunnel was less enlarged in the section with screw engagement in the β-TCP-containing screw group than the PLLA screw group. The addition of β-TCP to a degradable polymer such as PLLA creates an inorganic osteoconductive scaffold and changes the properties of the scaffold. During degradation, β-TCP breaks down into calcium ions and phosphates, which maintain an elevated pH around the implant [26–28]. This could act as a buffer to acidic degradation products of lactic acid or glycolic acid. These neutralizing effects around screws could result in less local reactivity or inflammatory responses, thereby reducing tunnel enlargement [29]. In the reconstructed oblique axial view on CT, bony integration was observed in the screw-bone contact area in the mid-tunnel area in the β-TCP-containing interference screw group. There was no gap between the adjacent native bone and implanted screw, and the screw tract was corticated as densely as cancellous bone (Fig. 4). Meanwhile, an enlarged tunnel and sclerotic margin without bony integration around the screw was observed in group A. Furthermore, no ingrowth of neo-bone tissue from the adjacent native bone was observed (Fig. 5). We attributed this to the presence of nondegradable poly L-lactide polymer, which could hinder the ingrowth of new bone from the native adjacent cancellous bone tissue.

**Fig. 6:**
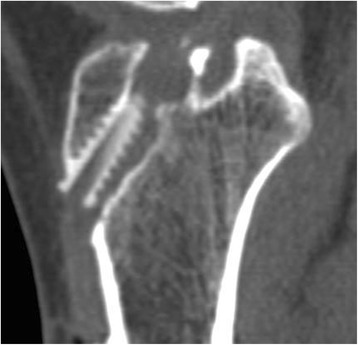
In group B, the degree of tunnel enlargement increased abruptly proximal to the screw end

Meanwhile, abruptly increased tunnel enlargement was observed in the proximal portion to β-TCP containing screw relative to a PLLA screw (Fig. 6). This conflicts with previous findings in patellar tendon autografts; bone tunnels in the tibia began to increase in size from the distal end [24]. Most probable explanation for this paradoxical enlargement is the cellular response to calcium phosphate (β-TCP). Particles proximally degraded from β-TCP by mechanical stress or synovial fluid influx likely activated osteoclasts and resulted in osteolysis, similar to the role of wear debris in aseptic loosening in arthroplasty. Many environmental factors are known to be involved in the osteointegration and degradation of calcium phosphate ceramics after implantation, including physiochemical processes and various cell activating molecules and cytokines [30–32].

Almost studies of explanted biodegradable interference screws have been overstated regarding to concerns about whether claims of bio-absorbability because of limited evidences of degradation [21, 33, 34]. It would be resulted from almost studies have been designed using patellar bone graft with little effect of container phenomenon with interference screw. However, when using hamstring grafts, the effects of the biodegradable interference screw on early tunnel widening must be considered. Despite these radiological observations, the clinical evaluation showed no significant difference, with good outcomes in both groups at the postoperative 2-year follow-up in current study. However, this could stem from the use of an additional extra-cortical screw with a spike washer for tibial aperture fixation.

Several studies have reported that use of β-TCP-containing screws reduces tunnel enlargement [13]. However, to the best of our knowledge, this is the first study to report the paradoxical tunnel enlargement in the proximal portion of tibial tunnel following fixation with the β-TCP-containing fixation screw. Further studies are needed to clarify the factors aggravating tunnel enlargement in ACL reconstruction.

The strength of this study is that to evaluate bone tunnel widening, immediate and postoperative 1- year CT scans were used instead of digital plan radiograph. Tunnel enlargement is believed to occur both intraoperatively and postoperatively. Intraoperative enlargement may be due to eccentric reaming, graft and reamer diameter mismatch, or use of the press-fit technique, so a difference in CSA between the reamer and actual tunnel is inevitable. In the current study, immediate postoperative CT images were used to obtain baselines values, so postoperative enlargement could be evaluated without intraoperative change bias. Furthermore, the tunnel was segmented into four different sections for more precise analysis; this enabled us to observe paradoxical enlargement in the area of the tunnel without screw engagement.

Nevertheless, the present study had some limitations. First, no histologic data were obtained to confirm complete degradation of the PLLA screws. Second, the follow-up period was relatively short. However, several studies have shown that most tunnel widening occurs during the first 3 months [35–39]. According to these studies, a one-year follow-up period is sufficient to observe enlargement of the tunnel itself, but tunnel remodeling and screw resorption are still occurring. A longer-term follow-up study is required to obtain a thorough understanding of tunnel enlargement and the fate of the implant and tunnel ossification. Third, the current study is limited by its small sample size. However, a post hoc power analysis was performed to determine whether the sample size had sufficient power. Fourth, we did not account for other confounding factors such as bone mineral density, screw and tunnel length ratio, or variations in tunnel location. However, there were no significant differences in age, gender, or BMI between the two groups of subjects. Another limitation is the lack of a comprehensive review of patients who underwent patellar tendon autograft ACL reconstruction. We investigated only the patients underwent DB-ACLR, might not the standard technique in some countries, not single-bundle method.

## Conclusion

Use of a β-TCP-containing interference screw in tibial aperture fixation reduced tunnel enlargement in the vicinity of the screw, whereas greater enlargement occurred proximal to the screw end relative to use of a pure PLLP interference screw. These paradoxical enlargements in use of β-TCP containing screws suggest that for reducing tunnel enlargement, the length of the interference screw should be as fit as possible with tunnel length in terms of using soft grafts.

## Additional file

Additional file 1: Table S-1. Results of Intraclass Correlation Coefficient (ICC) Value of Each Measurement. Interobserver reliability of CSAs at the four cutting levels ranged from 0.74 to 0.94. (DOCX 15 kb)

## Abbreviations

ACLR: anterior cruciate ligament reconstruction
AM: anteromedial
BMI: body mass index
BPTB: bone-patellar tendon-bone
CSA: cross-sectional area
CT: computed tomography
DB-ACLR: double bundle anterior cruciate ligament reconstruction
HSS: Hospital for Special Surgery
IKDC: International Knee Documentation Committee Score
PDS: polyparadioxanone
PGA: polyglycolic acid
PL: posterolateral
PLA: polylactic acid
PLLA: poly-L-lactic acid
β-TCP: β-tricalcium phosphate

## References

[1] McGuire DA, Barber FA, Elrod BF, Paulos LE (1999). Bioabsorbable interference screws for graft fixation in anterior cruciate ligament reconstruction. Arthroscopy.

[2] Barber FA (2000). Flipped patellar tendon autograft anterior cruciate ligament reconstruction. Arthroscopy.

[3] Drogset JO, Grontvedt T, Tegnander A (2005). Endoscopic reconstruction of the anterior cruciate ligament using bone-patellar tendon-bone grafts fixed with bioabsorbable or metal interference screws: a prospective randomized study of the clinical outcome. Am J Sports Med.

[4] Kaeding C, Farr J, Kavanaugh T, Pedroza A (2005). A prospective randomized comparison of bioabsorbable and titanium anterior cruciate ligament interference screws. Arthroscopy.

[5] Laxdal G, Kartus J, Eriksson BI, Faxen E, Sernert N, Karlsson J (2006). Biodegradable and metallic interference screws in anterior cruciate ligament reconstruction surgery using hamstring tendon grafts: prospective randomized study of radiographic results and clinical outcome. Am J Sports Med.

[6] Barber FA, Dockery WD (2006). Long-term absorption of poly-L-lactic acid interference screws. Arthroscopy.

[7] Ma CB, Francis K, Towers J, Irrgang J, Fu FH, Harner CH (2004). Hamstring anterior cruciate ligament reconstruction: a comparison of bioabsorbable interference screw and endobutton-post fixation. Arthroscopy.

[8] Warden WH, Chooljian D, Jackson DW (2008). Ten-year magnetic resonance imaging follow-up of bioabsorbable poly-L-lactic acid interference screws after anterior cruciate ligament reconstruction. Arthroscopy.

[9] Bourke HE, Salmon LJ, Waller A, Winalski CS, Williams HA, Linklater JM, Vasanji A, Roe JP, Pinczewski LA (2013). Randomized controlled trial of osteoconductive fixation screws for anterior cruciate ligament reconstruction: a comparison of the Calaxo and Milagro screws. Arthroscopy.

[10] Foldager C, Jakobsen BW, Lund B, Christiansen SE, Kashi L, Mikkelsen LR, Lind M (2010). Tibial tunnel widening after bioresorbable poly-lactide calcium carbonate interference screw usage in ACL reconstruction. Knee Surg Sports Traumatol Arthrosc.

[11] Moisala AS, Jarvela T, Paakkala A, Paakkala T, Kannus P, Jarvinen M (2008). Comparison of the bioabsorbable and metal screw fixation after ACL reconstruction with a hamstring autograft in MRI and clinical outcome: a prospective randomized study. Knee Surg Sports Traumatol Arthrosc.

[12] Doig GS, Simpson F (2005). Randomization and allocation concealment: a practical guide for researchers. J Crit Care.

[13] Carulli C, Matassi F, Soderi S, Sirleo L, Munz G, Innocenti M (2017). Resorbable screw and sheath versus resorbable interference screw and staples for ACL reconstruction: a comparison of two tibial fixation methods. Knee Surg Sports Traumatol Arthrosc.

[14] Ahn JH, Wang JH, Lee YS, Kim JG, Kang JH, Koh KH (2011). Anterior cruciate ligament reconstruction using remnant preservation and a femoral tensioning technique: clinical and magnetic resonance imaging results. Arthroscopy.

[15] Hanley ST, Warren RF (1987). Arthroscopic meniscectomy in the anterior cruciate ligament-deficient knee. Arthroscopy.

[16] Buss DD, Warren RF, Wickiewicz TL, Galinat BJ, Panariello R (1993). Arthroscopically assisted reconstruction of the anterior cruciate ligament with use of autogenous patellar-ligament grafts. Results after twenty-four to forty-two months. J Bone Joint Surg Am.

[17] Iorio R, Vadala A, Argento G, Di Sanzo V, Ferretti A (2007). Bone tunnel enlargement after ACL reconstruction using autologous hamstring tendons: a CT study. Int Orthop.

[18] Aga C, Wilson KJ, Johansen S, Dornan G, La Prade RF, Engebretsen L (2017). Tunnel widening in single- versus double-bundle anterior cruciate ligament reconstructed knees. Knee Surg Sports Traumatol Arthrosc.

[19] Nebelung W, Becker R, Merkel M, Ropke M (1998). Bone tunnel enlargement after anterior cruciate ligament reconstruction with semitendinosus tendon using Endobutton fixation on the femoral side. Arthroscopy.

[20] Hovis WD, Bucholz RW (1997). Polyglycolide bioabsorbable screws in the treatment of ankle fractures. Foot Ankle Int.

[21] Stahelin AC, Weiler A, Rufenacht H, Hoffmann R, Geissmann A, Feinstein R (1997). Clinical degradation and biocompatibility of different bioabsorbable interference screws: a report of six cases. Arthroscopy.

[22] Bergsma JE, de Bruijn WC, Rozema FR, Bos RR, Boering G (1995). Late degradation tissue response to poly(L-lactide) bone plates and screws. Biomaterials.

[23] Lajtai G, Noszian I, Humer K, Unger F, Aitzetmuller G, Orthner E (1999). Serial magnetic resonance imaging evaluation of operative site after fixation of patellar tendon graft with bioabsorbable interference screws in anterior cruciate ligament reconstruction. Arthroscopy.

[24] Lajtai G, Humer K, Aitzetmuller G, Unger F, Noszian I, Orthner E (1999). Serial magnetic resonance imaging evaluation of a bioabsorbable interference screw and the adjacent bone. Arthroscopy.

[25] Aga C, Rasmussen MT, Smith SD, Jansson KS, LaPrade RF, Engebretsen L, Wijdicks CA (2013). Biomechanical comparison of interference screws and combination screw and sheath devices for soft tissue anterior cruciate ligament reconstruction on the tibial side. Am J Sports Med.

[26] Kamitakahara M, Ohtsuki C, Miyazaki T (2008). Review paper: behavior of ceramic biomaterials derived from tricalcium phosphate in physiological condition. J Biomater Appl.

[27] Honda Y, Kamakura S, Sasaki K, Suzuki O (2007). Formation of bone-like apatite enhanced by hydrolysis of octacalcium phosphate crystals deposited in collagen matrix. J Biomed Mater Res B Appl Biomater.

[28] Okuda T, Ioku K, Yonezawa I, Minagi H, Kawachi G, Gonda Y, Murayama H, Shibata Y, Minami S, Kamihira S, Kurosawa H, Ikeda T (2007). The effect of the microstructure of beta-tricalcium phosphate on the metabolism of subsequently formed bone tissue. Biomaterials.

[29] Agrawal CM, Athanasiou KA (1997). Technique to control pH in vicinity of biodegrading PLA-PGA implants. J Biomed Mater Res.

[30] Braux J, Velard F, Guillaume C, Bouthors S, Jallot E, Nedelec JM, Laurent-Maquin D, Laquerriere P (2011). A new insight into the dissociating effect of strontium on bone resorption and formation. Acta Biomater.

[31] Penolazzi L, Lambertini E, Tavanti E, Torreggiani E, Vesce F, Gambari R, Piva R (2008). Evaluation of chemokine and cytokine profiles in osteoblast progenitors from umbilical cord blood stem cells by BIO-PLEX technology. Cell Biol Int.

[32] Lisignoli G, Toneguzzi S, Pozzi C, Piacentini A, Riccio M, Ferruzzi A, Gualtieri G, Facchini A (1999). Proinflammatory cytokines and chemokine production and expression by human osteoblasts isolated from patients with rheumatoid arthritis and osteoarthritis. J Rheumatol.

[33] McGuire DA, Barber FA, Milchgrub S, Wolchok JC (2001). A postmortem examination of poly-L lactic acid interference screws 4 months after implantation during anterior cruciate ligament reconstruction. Arthroscopy.

[34] Martinek V, Seil R, Lattermann C, Watkins SC, Fu FH (2001). The fate of the poly-L-lactic acid interference screw after anterior cruciate ligament reconstruction. Arthroscopy.

[35] Jarvela T, Moisala AS, Paakkala T, Paakkala A (2008). Tunnel enlargement after double-bundle anterior cruciate ligament reconstruction: a prospective, randomized study. Arthroscopy.

[36] Lee YS, Lee SW, Nam SW, Oh WS, Sim JA, Kwak JH, Lee BK (2012). Analysis of tunnel widening after double-bundle ACL reconstruction. Knee Surg Sports Traumatol Arthrosc.

[37] Siebold R, Cafaltzis K (2010). Differentiation between intraoperative and postoperative bone tunnel widening and communication in double-bundle anterior cruciate ligament reconstruction: a prospective study. Arthroscopy.

[38] Lind M, Feller J, Webster KE (2009). Bone tunnel widening after anterior cruciate ligament reconstruction using EndoButton or EndoButton continuous loop. Arthroscopy.

[39] Hoher J, Moller HD, Fu FH (1998). Bone tunnel enlargement after anterior cruciate ligament reconstruction: fact or fiction?. Knee Surg Sports Traumatol Arthrosc.

