# Supplementary material for: Paradoxical tunnel enlargement after ACL reconstruction with hamstring autografts when using β-TCP containing interference screws for tibial aperture fixation- prospectively comparative study
Source: BMC Musculoskelet Disord. 2017 Sep 16;18:398. doi: 10.1186/s12891-017-1757-0 (PMC5602947; doi:10.1186/s12891-017-1757-0)
Supplement: Additional file 1: Table S-1. — Results of Intraclass Correlation Coefficient (ICC) Value of Each Measurement. Interobserver reliability of CSAs at the four cutting levels ranged from 0.74 to 0.94. (DOCX 15 kb) [file 12891_2017_1757_MOESM1_ESM.docx]

**Additional file 1**

**Appendix**

Results of Intraclass Correlation Coefficient (ICC) Value of Each Measurement

|  | Cross Sectional Area | | | | | | | |
| --- | --- | --- | --- | --- | --- | --- | --- | --- |
|  | AM | | | | PL | | | |
|  | Joint line | Mid-tunnel | Mid-screw | Outlet | Joint line | Mid-tunnel | Mid-screw | Outlet |
| Intertester |  |  |  |  |  |  |  |  |
| ICC | 0.91 | 0.89 | 0.94 | 0.81 | 0.83 | 0.90 | 0.90 | 0.74 |
| Lower ICC | 0.85 | 0.86 | 0.90 | 0.70 | 0.74 | 0.84 | 0.80 | 0.61 |
| Upper ICC | 0.94 | 0.93 | 0.96 | 0.88 | 0.90 | 0.94 | 0.88 | 0.84 |
